# Supplementary material for: Effect of Radio-Chemotherapy on PD-L1 Immunohistochemical Expression in Head and Neck Squamous Cell Carcinoma
Source: J Pers Med. 2023 Feb 18;13(2):363. doi: 10.3390/jpm13020363 (PMC9965293; doi:10.3390/jpm13020363)
Supplement: Supplementary file 1 [file jpm-13-00363-s001.zip › jpm-2128062-supplementary.pdf]

Table S1. Search strategies.

Database	
Pubmed	
	#1 "head"[MeSH Terms] OR "head"[All Fields] OR ("hnsccs"[All Fields] OR "squamous cell carcinoma of head and neck"[MeSH Terms] OR ("squamous"[All Fields] AND "cell"[All Fields] AND "carcinoma"[All Fields] AND "head"[All Fields] AND "neck"[All Fields]) OR "squamous cell carcinoma of head and neck"[All Fields] OR "hnscc"[All Fields]) OR ("mouth"[MeSH Terms] OR "mouth"[All Fields] OR "oral"[All Fields]) OR ("tongue"[MeSH Terms] OR "tongue"[All Fields] OR "tongues"[All Fields] OR "tongue s"[All Fields]) OR ("mouth"[MeSH Terms] OR "mouth"[All Fields] OR "mouths"[All Fields] OR "mouth s"[All Fields] OR "mouthed"[All Fields] OR "mouthful"[All Fields] OR "mouthfuls"[All Fields] OR "mouthing"[All Fields]) OR ("nasalance"[All Fields] OR "nasality"[All Fields] OR "nasalization"[All Fields] OR "nasalized"[All Fields] OR "nasally"[All Fields] OR "nose"[MeSH Terms] OR "nose"[All Fields] OR "nasal"[All Fields] OR "nasals"[All Fields]) OR "sinonasal"[All Fields] OR "sino-nasal"[All Fields] OR "paranasal"[All Fields] OR ("pharynx"[MeSH Terms] OR "pharynx"[All Fields] OR "pharynxs"[All Fields] OR "pharynxes"[All Fields]) OR ("pharyngeals"[All Fields] OR "pharynges"[All Fields] OR "pharynx"[MeSH Terms] OR "pharynx"[All Fields] OR "pharyngeal"[All Fields]) OR ("oropharynx"[MeSH Terms] OR "oropharynx"[All Fields] OR "oropharynxes"[All Fields]) OR ("oropharynx"[MeSH Terms] OR "oropharynx"[All Fields] OR "oropharyngeal"[All Fields]) OR ("larynx"[MeSH Terms] OR "larynx"[All Fields] OR "larynxes"[All Fields]) OR ("larynges"[All Fields] OR "laryngitis"[MeSH Terms] OR "laryngitis"[All Fields] OR "laryngitides"[All Fields] OR "larynx"[MeSH Terms] OR "larynx"[All Fields] OR "laryngeal"[All Fields]) OR ("hypopharynx"[MeSH Terms] OR "hypopharynx"[All Fields] OR "hypopharynges"[All Fields] OR "hypopharynxes"[All Fields]) OR ("hypopharynx"[MeSH Terms] OR "hypopharynx"[All Fields] OR "hypopharyngeal"[All Fields]) OR ("vocal"[All Fields] OR "vocales"[All Fields] OR "vocalic"[All Fields] OR "vocalisation"[All Fields] OR "vocalisations"[All Fields] OR "vocalised"[All Fields] OR "vocalising"[All Fields] OR "vocalization"[All Fields] OR "vocalizations"[All Fields] OR "vocalize"[All Fields] OR "vocalized"[All Fields] OR "vocalizer"[All Fields] OR "vocalizers"[All Fields] OR "vocalizes"[All Fields] OR "vocalizing"[All Fields] OR "vocally"[All Fields] OR "vocals"[All Fields]) OR ("cone rod dystrophies"[MeSH Terms] OR ("cone rod"[All Fields] AND "dystrophies"[All Fields]) OR "cone rod dystrophies"[All Fields] OR "cord"[All Fields]) OR ("cord s"[All Fields] OR "cords"[All Fields])
	#2 "CD274"[All Fields] OR "B7-H"[All Fields] OR "B7H1"[All Fields] OR "PD-L1"[All Fields] OR "PDCD1LG1"[All Fields] OR "PDL1"[All Fields] OR "hPD-L1"[All Fields] OR "programmed cell death-ligand 1"[All Fields] OR "programmed cell death-ligand 1"[All Fields]
	#3 #1 AND #2
	#4 "B7-H1 Antigen"[MeSH Terms] OR "cd274 protein human"[Supplementary Concept]
	#5 "Squamous Cell Carcinoma of Head and Neck"[MeSH Terms]
	#6 #4 AND #5
	#3 OR #6
Embase	
	#1 'head'/exp OR head OR 'hnscc'/exp OR hnscc OR oral OR 'tongue'/exp OR tongue OR 'mouth'/exp OR mouth OR nasal OR sinonasal OR 'sino nasal' OR paranasal OR 'pharynx'/exp OR pharynx OR pharyngeal OR 'oropharynx'/exp OR oropharynx OR oropharyngeal OR 'larynx'/exp OR larynx OR laryngeal OR 'hypopharynx'/exp OR hypopharynx OR hypopharyngeal OR vocal OR cord OR cords
	#2 cd274 OR 'b7 h' OR b7h1 OR 'pd l1' OR pdcd1l1 OR pdcd1lg1 OR pdl1 OR 'hpd l1' OR 'programmed cell death-ligand 1' OR 'programmed cell death ligand 1'
	#3 #1 AND #2 AND [embase]/lim NOT [medline]/lim

Table S2. Summary of included studies.

Author, year (Country)	N cases	Sites	Therapy	Scoring system	Clone and platform	Main results	Other results and limitations
Clone E1L3N (6 studies)							
Delafoy, 2021 (France) [32]	44 (91% M, median age 65 y)	HNSCC NOS (Bx and Rx) (FU 24 months)	Concurrent cisplatin and RT	TC and IC scored separately, positive if >1%	E1L3N on Ventana Benchmark XT	• In 36 paired specimens before and after RT-CHT<br>• Statistically significant correlation between the PD-L1 on the initial and relapsing tumor, both on TC or IC (p = 0.001)<br>• Change of PD-L1 expression in 36.1%, among whom 7 (19.4%) had positivization on TC and negativization in 6 (16.7%) at recurrence<br>• Decrease in PD-L1 expression after radiation of 36.1% on TC and 19.4% on IC	• No prognostic value of PD-L1 expression (P>0.7) for OS and PFS<br>• No correspondence between Bx and Rx nor separation of data
Doescher, 2020 (Germany) [33]	67 (mean age 62 y)	HNSCC excluded sinonasal and rhinopharyngeal (Bx) (median FU 26 months)	Concurrent platinum-based CHT and RT	TC and IC scored separately with H-score	E1L3N on Dako Autostainer Universal Staining System	• No significant changes in the expression of PD-1 on neither tumor nor TIL during therapy<br>• No significant changes by therapy and no significant differences for PD-L1 expression on TC overall (p=0.28)<br>• PD-L1 expression on IC of non-responders was significantly lower before RT-CHT (p=0.008)	• Adverse prognostic value of PD-L1, PD-1 and CD27<br>• No use of established CPS cutoffs as in trials<br>• Small sample size<br>• Heterogeneity in tumor locations, fixation and storage of tissue blocks and treatments with no stratification
Gomez-Roca, 2020 (France) [34]	26 (81% M, median age 59.5 y)	Oral, OP, L, HP (Bx and Rx)	Inhibitor of apoptosis protein Debio 1143 with and without cisplatin	TC and IC as % positive cells	E1L3N on Dako Autostainer EnVision FLEX System	• Increased PD-L1 levels in immune cells with neoadjuvant target therapy with inhibitor of apoptose protein and in combination with cisplatin, but not with cisplatin alone<br>• PD-L1 levels remained at baseline levels of increased not significantly in TC	• Pharmacodynamic and pharmacogenomic study<br>• No use of established CPS cutoffs as in trials
Leduc, 2018 (France) [36]	21 (67% M, medina age 54 y)	Oral and L (Bx and Rx)	Docetaxel, platinum and fluorouracil (TPF)	TC and IC scored separately with >5% as positive	E1L3N on Ventana Discovery Ultra autostainer and HQ-amplification kit	• Significant increase of PD-L1 expression in IC after therapy (71%, p=0.003)<br>• Significant increase in PD-L1 expression also in TC after therapy (38%, p=0.005)	• Therapy also induce increase of CD8 TILs (p=0.01)<br>• Small sample size
Long, 2021 (China) [39]	24 (75% M, median age 47 y)	NP	Concurrent IMRT and cisplatin	TPS, positive if >1%, CPS	E1L3N, platform not stated, and 22C3 for imagery	• PD-L1 expression unchanged in 7 (29%), increased in 10 (42%), and decreased in 7 patients (29%)<br>• PD-L1 expression with CPS unchanged in 4 (17%), increased in 8	• PD-L1 expression correlated with better clinical response<br>• Not full correspondence of data in tables and text of the article

						(33%), and decreased in 12 patients (50%)	
Ock, 2017 (Korea) [44]	35 (89% M, mean age 63 y)	OP mainly	Docetaxel, platinum and fluorouracil (TPF) regimens or cisplatin-based regimens and concurrent RT	TPS>5% as positive	E1L3N on Ventana Benchmark XT	• Statistically significant up-regulation of PD-L1 in 69% of originally negative cases (p=0.003)<br>• 18% of positive cases were negative after therapy, with no statistical significance (p=0.72)<br>• Radiotherapy significantly induces up-regulation of PD-L1 (p<0.001)	• Small sample size<br>• Down-regulation of EMT markers after therapy<br>• Unclear detailing of IHC protocol
Clone SP142 (2 studies)							
Chan, 2017 (China) [36]	161 (73% M, median age 53 y)	NP	IMRT and CHT with cisplatin	TC and IC with cutoff 1%	SP142 on Ventana Benchmark	• Reduction of PD-L1 expression in 56% cases in IC and in 33% in TC after treatment<br>• Median decrease lower than 3%<br>• 47% positive cases changed to negative, 12% became positive at cutoff 1%<br>• No influence of type of therapy	• No separated data for patients receivinfg radiotherapy alone<br>• Similar changes for CD8<br>• No correlation of PD-L1 with OS or PFS
So, 2020 (Korea) [45]	42 (74% M, mean age 59.5 y)	Oral cavity, L, OP, HP, sinuses (Bx) (FU 61 months)	RT only	TC and IC positive if >10%, automated scoring on WSI digitized slides	SP142 on Ventana Benchmark XT with Optiview DAB detection kit and Optiview Amplification kit	• Ratio of recurrent (R) vs initial (I) PD-L1 expression was <1 in 69% cases with TC turning negative in 23% and positive in 9%<br>• Similar results for ratio of CD8 TILs	• No prognostic role of PD-L1 decrease, while patients with decrease of CD8 TILs showed worse prognosis (p=0.003)<br>• Reported limitations: small sample size, retrospective design, and different treatment characteristics among patients
Other clones (7 studies, a clone each)							
Karabajakian, 2021 (France) [35]	35 (71% M, median age 64 y)	Oral, OP, HP, L (Bx and Rx)	RT-CHT NOS	CPS	QR1 on Ventana Ultra View	• At CPS1 and CPS20 64% and 67% concordance between initial and recurrent tumor<br>• Majority of samples positive at CPS1 remained positive (76%), while majority of negative CPS samples at diagnosis became positive at relapse (75%)<br>• At CPS20 majority of negative CPS samples at diagnosis remained negative at relapse (71%)	• No special limitations; additional strong correlation between gene expression profile of PD-L1 with mRNA and CPS scoring
Naruse, 2020 (Japan) [41]	121 (76% M)	Tongue	Platinum-based CHT	TC only, positive if >5%	Abcam ab156361	• No marked difference in expression of PD-L1 in recurrent patients who had surgery alone or CHT plus surgery<br>• Higher expression in recurrent patients treated with CHT alone<br>• Lower expression in CHT treated patients without local recurrence	• No use of CPS or approved assay<br>• No reporting of CI or p values for comparisons; only reporting for correlation with clinicopathological variables and prognosis<br>• No adjustments reported nor FU time

Ono, 2020 (Japan) [42]	30 (90% M, mean age 66 y)	L, OP, HP	Cisplatin-based RT-CHT	TPS and IC density score	clone D3 on Ventana BenchMark Ultra with Ultraview detection kit	• No significant changes in PD-L1 expression after RT-CHT (p>0.41)<br>• Same results for PD-L2 and other immune markers<br>• Positive correlation between increased CD8 TILs and PD-L1 up-regulation after therapy (p=0.007)	• Small sample size
Pflumio, 2021 (France) [37]	100 (79% M, mean age 62.4 y)	Oral, OP, L, HP (Rx) (FU median 18 months)	RT-CHT NOS	TPS>1%	SP263	• CD3+ TIL significantly lower in intratumoral and stromal components in the irradiated area cohort (p=0.001)<br>• No significant difference between CD8+ TIL in the two cohorts (p=0.273)<br>• Significantly lower percentage of PD-L1+ TC within the irradiated area cohort than the de novo cohort (p=0.001)<br>• Significantly fewer tumors with PD-L1+ IC in the irradiated area cohort (p=0.01)	• Indirect comparison, as 50 irradiated cancers are compared with 50 de novo cancers<br>• No CPS used
Seki-Soda, 2021 (Japan) [43]	71 (68% M)	Oral cavity (Bx and Rx)	Preoperative fluorouracil-based CHT	TC	28-8, platform not stated	• PD-L1 positivity in TC significantly decreased by CHT irrespective of clinical response	• No use of established CPS cutoffs as in trials
Shen, 2020 (China) [40]	47 (100% M, median age 64 y)	HP (Bx and Rx)	Platinum-based CHT and RT	TC	66248-1-Ig, Proteintech, platform not stated	• PD-L1 expression increased after RT	• Not stated a clear cutoff for positivity, only 10% for low vs high<br>• Only 25 cases with available pre and post RT samples
Wolf, 2020 (USA) [46]	96 (72% M, mean age 60.2 y)	Oral cavity (bx and Rx)	Multi-cytokine preparation with cyclophosphamide	IC and H-score, CPS	Multiplex fluorescence immunohistochemistry	• No significant changes in PD-L1 expression after CHT<br>• Trend to positivization in 38% of negative cases before CHT with add of multicytokine preparation	• Small sample size (only 28 paired specimens for complete analysis)

Bx, biopsy specimen; CHT, chemotherapy; CI, confidence interval; CPS, combined positive score; EMT, epithelia-mesenchymal transition; FU, follow-up; HNSCC, head and neck squamous cell carcinoma; HP, hypopharynx; IC, immune cells; IHC, immunohistochemistry; IMRT, intensity modulated radiation therapy; L, laryngeal; M, male; NOS, not otherwise specified; NP, nasopharyngeal; OP, oropharyngeal; OS, overall survival; PFS, progression-free survival; RP, rhinopharynx; RT-CHT, radio-chemotherapy; Rx, resection specimen; TC, tumor cells; TILs, tumor-infiltrating lymphocytes; TPS, tumor proportion score; WSI, whole-slide imaging; y, years.
